# Staphylococcus hemolyticus: The Most Common and Resistant Coagulase-Negative Staphylococcus Species Causing Bacteremia in North India

**DOI:** 10.7759/cureus.51680

**Published:** 2024-01-04

**Authors:** Anuragani Verma, Sanjay Kumar, Vimala Venkatesh, Parul Jain, RajKumar Kalyan, Himanshu Reddy

**Affiliations:** 1 Microbiology, King George's Medical University, Lucknow, IND; 2 Internal Medicine, King George's Medical University, Lucknow, IND

**Keywords:** infection, blood culture, bacteraemia, staphylococcus hemolyticus, coagulase-negative staphylococcus

## Abstract

Introduction: Coagulase-negative *Staphylococcus *(CoNS) species are normal skin commensals but may also cause bacteremia. Therefore, isolating a CoNS species on blood culture often leads to a diagnostic dilemma about whether to consider the isolate as a true pathogen or not. This study was done to understand the distribution of various CoNS species in bloodstream infections, determine their antibiotic resistance patterns, and identify possible risk factors and patient outcomes in hospital settings.

Materials and methods: Inpatients with confirmed bacteremia defined as isolation of the same CoNS species with similar antibiograms from paired blood culture bottles, which were obtained from patients with at least clinical evidence of infection, were included. The isolates obtained were studied for CoNS species distribution and antibiotic resistance patterns, and the corresponding patients were assessed for possible risk factors and outcomes.

Results: A total of 170 CoNS isolates obtained from 85 patients were analyzed. *Staphylococcus* *haemolyticus *(*S. haemolyticus*)(90, 52.9%) was the most common species isolated, and it was also the most resistant of all, followed by *S. hominis* (50, 29.4%), *S. epidermidis* (26, 15.3%), *S. lentus* (2,1.2%), and *S. succinus* (2,1.2%). *S. haemolyticus* and *S. hominis* were significantly more isolated from patients aged 18-60 years and >60 years, respectively. Methicillin-resistant (MR)-CoNS (68.8%) were significantly more resistant than methicillin-sensitive (MS)-CoNS (31.2%) to certain antibiotics, and none were resistant to vancomycin, linezolid, or teicoplanin. Mortality occurred in 17.6% of patients, which was most commonly associated with *S. haemolyticus* infection.

Conclusion: Age-specific predisposition of CoNS species, high rates of methicillin resistance, and mortality in CoNS bacteremia are highlights of this study. To our knowledge, we are the first to study the age-related association of CoNS species.

## Introduction

Coagulase-negative *Staphylococcus *species (CoNS) were earlier regarded as a contaminant of clinical specimens because it is a commensal on skin and mucous membranes. But, over the last two decades, its clinical significance has increasingly been understood and is now an established cause of bacteremia. Guidelines exist for differentiating true bacteremia from contamination, both clinically and microbiologically [1-3]. The surge in the incidence of CoNS bacteremia may be due to the pool of susceptible populations available, such as premature infants, chronically ill, morbid, elderly patients, and immune-compromised patients, as well as the increased use of antibiotics and foreign bodies such as indwelling and insertion devices [4].

A plethora of studies are available on CoNS bacteremia, but most of them deal with CoNS as a whole and do not differentiate between species, which is essential for epidemiological analysis, patient management, and formulating infection prevention and control practices [5, 6]. Even less virulent CoNS species can cause infections, especially in the presence of favorable conditions like foreign bodies. Evidence suggests that certain strains and species of *Staphylococcus epidermidis *(*S. epidermidis*) have unique virulence factors, such as the insertion sequence (IS256) and novel arginine catabolic mobile elements [5,7,8]. Hence, constant vigilance of species causing bacteremia and the associated risk factors is essential.

Furthermore, increasing antibiotic resistance is a concern with CoNS, as is for other nosocomial pathogens, limiting the therapeutic option [4]. However, CoNS bacteremia is generally thought to be associated with low mortality [9, 10]. Therefore, this research aimed to ascertain the patterns of antibiotic resistance across different CoNS species in bloodstream infections, as well as to investigate potential risk factors and patient outcomes in hospital settings.

## Materials and methods

This was a hospital-based observational study conducted in the department of microbiology at King George’s Medical University, Lucknow, India, during a period extending from September 2019 to March 2020. Ethical clearance for the study was obtained from the institutional ethics committee (approval number: 1046/Ethics/19, dated July 29, 2019).

The inclusion criteria for the study were: 1) patients with suspected bloodstream infections for whom paired blood culture bottles were received; 2) both culture bottles yielded the same species of CoNS on the BacT/Alert (bioMerieux, Durham, NC) automated blood culture system; 3) both CoNS isolates had the same antibiotic susceptibility pattern by the Kirby-Bauer disc diffusion method; and 4) patients had at least one clinical evidence of infection from among the following four criteria: fever >38.0°C or hypothermia <36.0°C, tachycardia >90 beats/minute, tachypnea >20 breaths/minute, leucocytosis >12x109/l or leucopenia <4x109/l. These criteria were based on those described by the Centers for Disease Control and Prevention, USA, for bacteremia [11].

The exclusion criteria for the study were: 1) patients with suspected bloodstream infections for whom single blood culture bottles were received; 2) both culture bottles yielded the different species of bacterial isolates on the BacT/Alert (bioMerieux) automated blood culture system; 3) both CoNS isolates had a different antibiotic susceptibility pattern as determined by the Kirby-Bauer disc diffusion method; and 4) patients had no clinical evidence of bloodstream infection.

The CoNS isolates meeting the above criteria were speciated using matrix-assisted laser desorption ionization-time of flight mass spectrometry (MALDI-TOF) (MALDI Biotyper, Bruker Daltonics, Leipzig, Germany), and their antibiogram was obtained by the Kirby-Bauer disc diffusion method using the following antibiotics: amikacin, gentamicin, clindamycin, erythromycin, cotrimoxazole, levofloxacin, ciprofloxacin, tetracycline, penicillin, teicoplanin, and linezolid. Vancomycin was tested by the minimum inhibitory concentration (MIC) method. Methicillin-resistant (MR)-CoNS were detected using a cefoxitin disc (Becton Dickinson, Franklin Lakes, NJ) test. Their spatial distribution within the hospital (wards from where they were isolated) was also studied with the application of stringent observance and subsequent measures, such as tracking illness transmission. Under the hospital-associated infection surveillance program, we implement focused measures, assign resources, and assess efficacious corrective action. Demographic data and follow-up data until discharge or death were obtained for these patients to study risk factors and outcomes.

Statistical analysis for the study was done using Prism software, version 6 (GraphPad Software, La Jolla, CA). Fischer’s exact test and Mann-Whitney U test were used to calculate the significance of the difference for categorical and non-parametric data, respectively. A two-tailed p-value of <0.05 was considered significant.

## Results

A total of 85 patients met the inclusion criteria and were hence included in the study. The CoNS species were most commonly isolated from patients aged 18-60 years (45, 52.9%), though 32.9% (n = 28) isolates were obtained from patients with extreme ages. A statistically significant association was observed between the CoNS species isolated and age. *Staphylococcus haemolyticus* and *S. hominis* were significantly more isolated from patients aged 18-60 years and >60 years, respectively (p-values: 0.001 and 0.0001, respectively). The male-to-female ratio of patients with CoNS infection was 1.8:1. No significant association of CoNS species with hospital area was observed; overall, the percentages of CoNS isolation were similar in the intensive care units and the general wards (35.2% and 37.6%, respectively) and were greater than those seen in other wards (27.1%). Since two isolates were obtained from each patient, a total of 170 isolates were obtained from 85 patients, and biotyping with MALDI-TOF revealed that *S. haemolyticus* was the most common species (90, 52.9%) isolated, followed by *S. hominis* (50, 29.4%) and *S. epidermidis* (26, 15.3%). *Staphylococcus lentus* and *S. succinus* were rarely isolated (2, 1.2% each) (Table 1).

**Table 1:** Demographic distribution and outcome of patients with different CoNS species CoNS: coagulase-negative *Staphylococcus*

Variables	N (%)	*Staphylococcus haemolyticus* n=45 (%)	p-value	*Staphylococcus* *hominis n*=25 (%)	p-value	*Staphylococcus* *epidermidis *n=13 (%)	p-value
Age distribution							
<5 years	4 (4.7)	1 (2.2)	0.001	2 (8)	0.0001	1 (7.7)	0.56
5-<18 years	12 (14.1)	6 (13.3)		1 (4)		4 (30.8)	
18-60 years	45 (52.9)	32 (71.1)		4 (16)		8 (61.5)	
>60 years	24(28.2)	6 (13.3)		18 (72)		0 (0)	
Sex distribution							
Males	55(64.7)	25 (45.6)	0.07	19 (76)	0.21	10 (76.9)	0.52
Females	30(35.2)	20 (44.4)		6 (24)		3 (23.1)	
Hospital distribution							
Intensive care units	30(35.2)	19 (42.2)	0.38	5 (20)	0.59	5 (38.5)	0.17
General wards	32(37.6)	17 (37.8)		12 (48)		2 (15.4)	
Others	23(27.1)	9 (20)		8 (32)		6 (46.2)	
Outcome							
Mortality	15 (17.6)	10 (22.2)	0.269	2 (8)	0.34	2 (15.4)	1.0

Both isolates of *S. lentus* were isolated from a 22-year-old female patient admitted to the ICU and were methicillin-resistant. A nine-year-old male patient admitted to a general ward was the source of both methicillin-sensitive *S. succinus* isolates. Since there were only two isolates for each of the latter two species, they were not considered for further analysis. The antibiotic susceptibility test performed on these isolates showed that the antibiogram varied with CoNS species. Of the three species, *S. haemolyticus* was found to have the maximum resistance to antibiotics, followed by *S. hominis*, and *S. epidermidis* had the least resistance. When individual antibiotics were considered, *S. hominis* was significantly more resistant to tetracycline (p-value: 0.05), and *S. epidermidis* was significantly less resistant to amikacin (p-value: 0.05), ciprofloxacin (p-value: 0.03), and erythromycin (p-value: 0.0001). A high proportion of cefoxitin resistance was observed among all the CoNS species (89.4%), though there was no significant difference among the species. Of the total isolates, 68.8% were MR-CoNS, and only 31.2% were methicillin-sensitive (Table 2).

**Table 2:** Antibiotic resistance pattern of various CoNS species CoNS: coagulase-negative *Staphylococcus*

Antibiotics	Total resistant isolates (n=170)	Resistant *Staphylococcus* *haemolyticus *isolates n=90 (%)	Resistant *Staphylococcus* *hominis *isolates n=50(%)	Resistant *Staphylococcus* *epidermidis *isolates n=26 (%)	Resistant *Staphylococcus* *lentus *isolates n=2	Resistant *Staphylococcus* *succinus *isolates n=2	p-value
Cefoxitin	118 (69.4)	64 (71.1)	38 (76)	14 (53.8)	2(100)	0(0)	0.95
Amikacin	18 (10.6)	14 (15.5)	4 (8.0)	0(0)	0(0)	0(0)	0.05
Gentamicin	40 (35.3%)	34 (37.8)	18 (36.0)	6 (23.1)	2 (100)	0(0)	0.2
Clindamycin	92 (54.1%)	50 (55.5)	28 (56.0)	12 (46.1)	2 (100)	0(0)	0.65
Erythromycin	121 (71.8)	80 (88.9)	40 (80.0)	0(0)	2 (100)	0(0)	
Cotrimoxazole	122 (71.8)	66 (73.3)	36 (72.0)	18 (69.2)	2 (100)	0(0)	0.95
Levofloxacin	60 (35.3)	32 (35.5)	22 (44.0)	6 (23.1)	0(0)	0(0)	0.35
Ciprofloxacin	88 (49.4)	50 (55.5)	32 (64.0)	6 (23.1)	0(0)	0(0)	
Tetracycline	22 (13.0)	8 (8.9)	12 (24.0)	2 (7.7)	0(0)	0(0)	0.05
Penicillin	152 (89.4)	76 (84.4)	46 (92.0)	26 (100)	2 (100)	2 (100)	0.9
Vancomycin	0 (0)	0 (0)	0 (0)	0 (0)	0 (0)	0 (0)	
Teicoplanin	0 (0)	0 (0)	0 (0)	0 (0)	0 (0)	0 (0)	
Linezolid	0 (0)	0 (0)	0 (0)	0 (0)	0 (0)	0 (0)	

Overall, MR-CoNS was significantly more resistant than methicillin-sensitive (MS)-CoNS to gentamicin, clindamycin, erythromycin, ciprofloxacin, and tetracycline. None of the isolates were resistant to vancomycin, linezolid, or teicoplanin (Table 3).

**Table 3:** Comparison of the antibiotic resistance pattern between MR-CoNS and MS-CoNS

Antibiotics	Methicillin-resistant coagulase-negative *Staphylococcus *(MR-CoNS) (n = 118, 68.8%)	Methicillin-sensitive coagulase-negative *Staphylococcus *(MS-CoNS) (n = 52, 30.6%)	p-value
Amikacin	14 (11.9)	4 (7.7)	0.564
Gentamicin	52 (44.1)	6 (11.5)	0.004
Clindamycin	78 (66.1)	16 (30.8)	0.003
Erythromycin	110 (93.2)	30 (57.7)	<0.001
Cotrimoxazole	86 (72.9)	38 (73.1)	0.985
Levofloxacin	46 (39.0)	10 (19.2)	0.074
Ciprofloxacin	74 (62.7)	8 (15.4)	<0.001
Tetracycline	20 (16.9)	0 (0)	0.025
Penicillin	110 (93.2)	42 (80.8)	0.124

Mortality occurred in 17.6% (n = 15) patients with CoNS bacteremia, which was most commonly observed in patients with *S. haemolyticus *infection (22.2%), though no significant association of any CoNS species with mortality was found (Table 1). No risk factor was associated with the outcome, but body temperature >38 °C or <36 °C was significantly associated with mortality (Table 4).

**Table 4:** Risk factors associated with mortality in patients with CoNS bacteremia CoNS: coagulase-negative *Staphylococcus*

Risk factors/ Signs and symptoms	Death (n=15)	Discharge (n=70)	p-value
Body temperature (>38ºC or < 36ºC)	14 (93.3)	39 (55.7)	0.016
Tachycardia	3 (20)	18 (28.1)	0.521
Tachypnea	6 (40)	14 (20)	0.146
Total leukocyte count (TLC) (>12,000/uL or <4,000/uL)	11 (73.3)	36 (51.4)	0.225
Diabetes	4 (26.7)	13 (18.6)	0.589
Malignancy	1 (5.8)	16 (22.9)	0.119
Immunosuppression	2 (13.3)	15 (21.4)	0.391
Recent surgery (within days)	0 (0)	3 (4.2)	1.00
Intravenous catheter	15 (100)	64 (91.4)	
Central line catheter	6 (40)	17 (24.3)	0.303

## Discussion

Isolation of CoNS species from blood culture often leads to a diagnostic dilemma: whether to consider it a true pathogen or a commensal. To be sure that only the true bloodstream pathogens are considered, stringent inclusion criteria were followed in this study, and only persons fulfilling the laboratory and clinical criteria for CoNS, as described by the National Healthcare Safety Network, were included. In the present study, 85 such patients were identified, and it was found that *S. haemolyticus* is the most common species isolated from bloodstream infections, followed by *S. hominis* and *S. epidermidis.* *Staphylococcus lentus* and *S. succinus* were rarely isolated; these isolates were not co-founders in the present study, and patients who had other pathogens on culture were excluded from this study [11]. Studies have shown that *S. epidermidis* is the most common cause of CoNS bacteremia in the Western world, and *S. haemolyticus* has emerged as the second most common species [12, 13].

A recent study from north India identified that the majority of CoNS isolated were constituted by *S. haemolyticus* (47.5%), followed by *S. epidermidis* (33.9%), *S. hominis* (11.86%), *S. cohnii *(5.08%), and *S. warneri* (1.69%) [14]. This emerging epidemiology is of concern, as *S. haemolyticus*, which is known to exhibit a higher MIC value for glycopeptides (vancomycin), is gradually replacing *S. epidermidis*, which in turn is an indolent species. This is also shown in the present study, where *S. haemolyticus* was more resistant to all classes of antibiotics (except vancomycin, teicoplanin, and linezolid), followed by *S. hominis*, and *S. epidermidis* showed the least resistance [15, 16].

Therefore, it is essential to monitor the prevalence of CoNS species that will help design tailored empirical therapy, for example, including amikacin, ciprofloxacin, and erythromycin for *S. epidermidis* and avoiding tetracycline for *S. hominis* at our center. Significant associations between age and CoNS species were found, meaning that in an 18- to 60-year-old patient with CoNS bacteremia, there is a high likelihood of isolating *S. haemolyticus*. Similarly, in patients over 60 years old, *S. hominis* is more likely to be the cause of CoNS bacteremia. These data will be useful for establishing age-appropriate empirical therapy.

To the best of our knowledge, this is the first study showing a relationship between age and predominant CoNS species and needs further evaluation. A high proportion of the CoNS species isolated in this study were methicillin-resistant (69.4%), which is even higher than the proportion of methicillin-resistant *S. aureus* (54%) causing bloodstream infections in previous Indian studies [15].

High drug resistance was observed in MR-CoNS as compared to the methicillin-sensitive species; the difference was statistically significant for gentamicin, clindamycin, erythromycin, ciprofloxacin, and tetracycline. Inducible clindamycin resistance was checked by the D-zone test. The only options left for the treatment of MR-CoNS are vancomycin, linezolid, and teicoplanin, for which none of the isolates were found to be resistant. However, as vancomycin resistance among CoNS has already been observed in India, these medications should be used cautiously and avoided if any other medication is sensitive to antibiotic susceptibility testing [16, 17].

On the other hand, several options, like gentamicin, clindamycin, erythromycin, ciprofloxacin, and tetracycline, are available for the treatment of MS-CoNS. A previous study conducted in patients with CoNS bacteremia reported 12.8% associated mortality rates. In a cohort study, mortality occurred in only 4.3% of cancer patients with febrile neutropenia who also had CoNS bacteremia [9]. A study on CoNS sepsis in very low birth weight infants reported no mortality at all [10]. In contrast to these studies, the present study reported mortality in a higher proportion of patients (17.6%), which might be due to the high prevalence of multidrug-resistant *S. haemolyticus* in the study population.

There are certain limitations of the study, primarily that the sample size was small and confined to a single center, and secondly, there was no molecular analysis to characterize the multidrug-resistant pattern of *S. haemolyticus*.

## Conclusions

The highlights of this study are the inclusion of cases of only true bacteremia, the study of age-specific predispositions of CoNS species, the high rates of methicillin resistance and mortality in patients with CoNS bacteremia, and the identification of *S. haemolyticus* as the most common and resistant species of CoNS causing true bacteremia in Indian hospital settings. It establishes the basis for future research into the reasons for the geographic variation in the most prevalent CoNS species that cause bacteremia.
